# Do Old Age and Comorbidity *via* Non-Communicable Diseases Matter for COVID-19 Mortality? A Path Analysis

**DOI:** 10.3389/fpubh.2021.736347

**Published:** 2021-11-16

**Authors:** Gour Gobinda Goswami, Mausumi Mahapatro, A. R. M. Mehrab Ali, Raisa Rahman

**Affiliations:** ^1^Department of Economics, North South University, Dhaka, Bangladesh; ^2^Department of History, Politics and Political Economy, Regis University, Denver, CO, United States; ^3^Aureolin Research, Consultancy and Expertise Development (ARCED) Foundation, Dhaka, Bangladesh

**Keywords:** COVID-19, death rate, mortality, determinants of COVID-19 mortality, path analysis, old age, comorbidity, non-communicable diseases

## Abstract

This paper used Our World data for coronavirus disease-2019 (COVID-19) death count, test data, stringency, and transmission count and prepared a path model for COVID-19 deaths. We augmented the model with age structure-related variables and comorbidity *via* non-communicable diseases for 117 countries of the world for September 23, 2021, on a cross-section basis. A broad-based global quantitative study incorporating these two prominent channels with regional variation was unavailable in the existing literature. Old age and comorbidity were identified as two prime determinants of COVID-19 mortality. The path model showed that after controlling for these factors, one *SD* increase in the proportion of persons above 65, above 70, or of median age raised COVID-19 mortality by more than 0.12 *SD*s for 117 countries. The regional intensity of death is alarmingly high in South America, Europe, and North America compared with Oceania. After controlling for regions, the figure was raised to 0.213, which was even higher. For old age, the incremental coefficient was the highest for South America (0.564), and Europe (0.314), which were substantially higher than in Oceania. The comorbidity channel *via* non-communicable diseases illustrated that one *SD* increase in non-communicable disease intensity increased COVID-19 mortality by 0.132 for the whole sample. The regional figure for the non-communicable disease was 0.594 for South America and 0.358 for Europe compared with the benchmark region Oceania. The results were statistically significant at a 10% level of significance or above. This suggested that we should prioritize vaccinations for the elderly and people with comorbidity *via* non-communicable diseases like heart disease, cancer, chronic respiratory disease, and diabetes. Further attention should be given to South America and Europe, which are the worst affected regions of the world.

## Highlights

- Using a cross-section path model for 117 countries, we found that one *SD* increase in old age concentration raised COVID-19 death by more than 0.12 *SD*s for the whole sample.
- One *SD* increase in non-communicable disease intensity led to 0.132 *SD* increases in COVID-19 death for the whole sample.
- The pandemic is causing disastrous effects mainly in South America and Europe, followed by North America compared with Oceania.
- This was a broad-based global study that incorporated a large number of countries by using secondary head-count data from Our World in Data.
- Vaccination drive, development assistance, and cooperation should be directed more toward older persons and people who have comorbidity from non-communicable diseases like heart disease, cancer, chronic respiratory disease, and diabetes.

## Introduction

**Figure 1:**
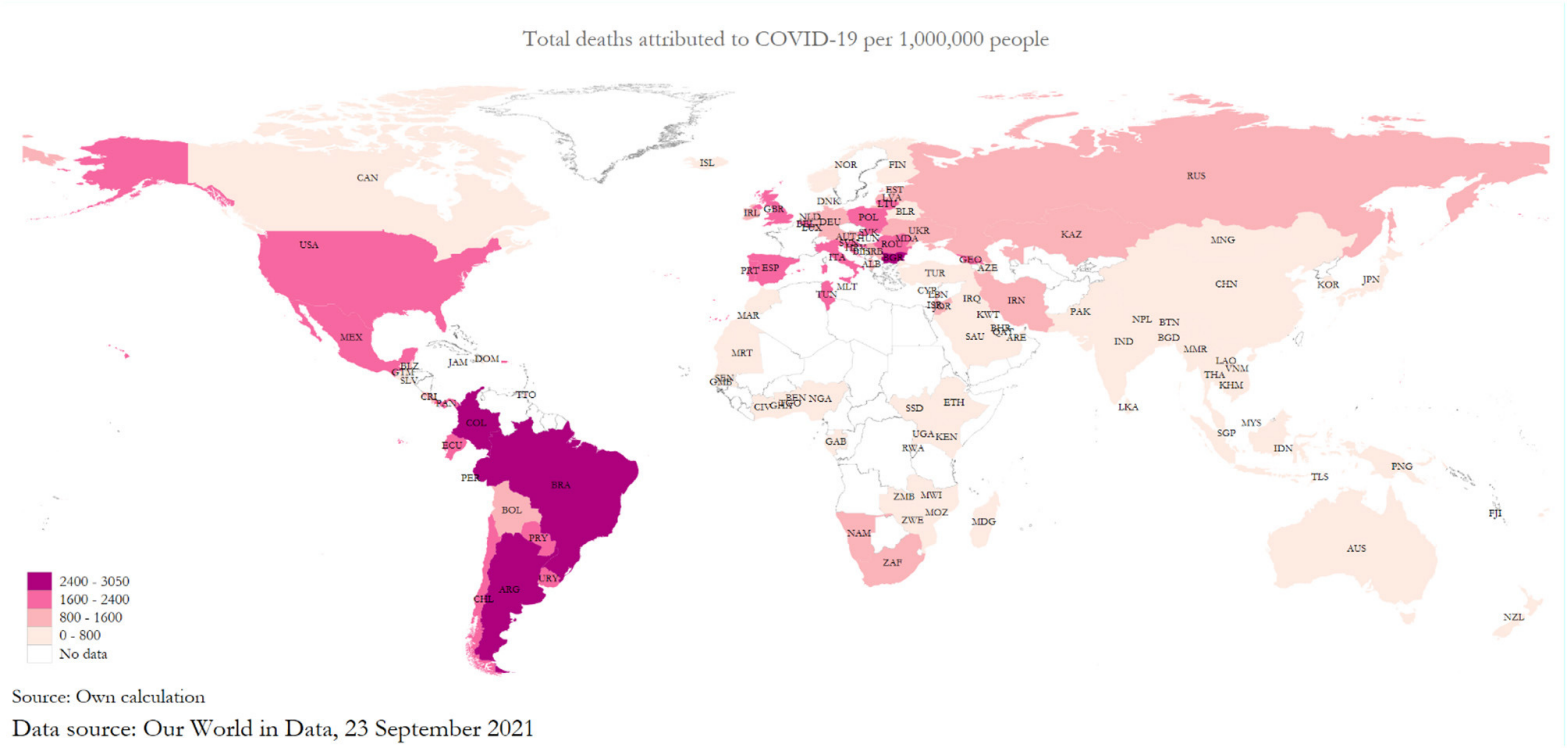
The global death pattern of coronavirus disease-2019 (COVID-19).

If we consult the coronavirus disease-2019 (COVID-19) death map for the world for September 23, 2021, we can observe that the intensity of COVID-19 is concentrated within South America, and Europe followed by North America with minimal figures for Africa, Asia, and Oceanian countries (Figure 1)^1^. What accounts for this variation in mortality across countries remains a puzzle with many pieces that need to be fitted. A large body of literature has gradually evolved to examine the possible determinants of COVID-19 death rate or mortality without consistent or conclusive results. There are several reasons for this, including countries or regions having their country-specific determinants and the virus mutating so fast that scientists and policymakers are hard-pressed in tracking its nature. Vaccination drives have commenced but to varying levels and degrees owing to differential access across countries. Apart from medical reasons, there are many socio-economic, demographic, cultural, environmental, and ecological reasons. The cause is also extended to political, institutional, and governance channels. Some country-based studies have identified old age hypothesis and comorbidity without accounting for the global determinants or regional variations. Most death rate modeling uses multiple regression frameworks or simple hypothesis tests without much control for interdependencies among test, transmission, stringency, and endogeneity. To fill this gap, we prepared a general mortality model for COVID-19 death in line with Goswami et al. (1), and developed it in the context of death rate modeling, and added old age and comorbidity as additional determinants of mortality. We used a global data set for 117 countries updated on September 23, 2021, and constructed two different models of COVID-19 death. These models were old age hypothesis and comorbidity or mortality *via* non-communicable diseases like heart diseases, cancer, respiratory illness, and diabetes to show their impacts on COVID-19 mortality in general and for six regions of the world. With this end in view, the paper was organized as follows: Section Literature Review provided a review of the literature; Section Data and Preliminary Methods described the data and variables used for the study; Section Path Model for COVID-19 Death developed a path model; Section Estimated Results and Interpretations provided a detailed analysis of the estimation results; Section Further Discussion provided further discussion; Section Conclusion and Policy Suggestions concluded with policy suggestions.

## Literature Review

The broad literature of COVID-19 can be classified into three types: identifying the determinants of death rate or mortality (2–8), identifying the determinants of transmission (1, 9–12), and identifying the economic, social, environmental and ecological impact of COVID-19 (12–22). However, these are primarily socio-economic studies related to COVID-19, which do not include a vast medical literature that focuses on the scientific causes, vulnerability, and overall health impact.

The existing studies indicate the various range of factors that contribute to the COVID-19 mortality which includes gender (23, 24), hesitancy of being vaccinated (15, 25), age (6, 10, 21, 22, 26–33), environmental, demographic factors, population density, biological and healthcare related factors (2, 3, 16), race (12, 18–20, 34), international travel (17), and pre-existing morbidity (7–9, 35–38).

If we examine the existing literature, we can assert that a broad-based socio-economic study explaining the determinants of COVID-19 covering more than 100 countries of the world is non-existent. Most of the existing studies in this area use multiple regression techniques or conduct hypothesis tests using data from different hospitals, countries, or county levels. COVID-19 variables are potentially interdependent, and they follow a complicated structure ranging from economy, science, demography, governance, and other factors. For example, testing depends on the overall economic capacity of countries. In addition, it also depends on the stringency situation of a country and ability which can also be used as an instrument for identifying the transmission history (1). After taking all these channels into account, transmission impacts the COVID-19 death rate. This complex mechanism must be addressed in preparing a suitable model for COVID-19 death. This is the research gap that we address in this study.

Our paper has identified two prime determinants of COVID-19 related mortality, namely old age and non-communicable disease-related comorbidity. Various studies have attempted to show the effect of old age and non-communicable disease-related comorbidity on COVID-19 mortality using multivariable logistic regression analysis, Cox proportional hazards models, Watts–Strogatz model, Meta-analysis, random-effects models, Cochran's Q test and sensitivity analysis, Kaplan-Meier method, Kolmogorov-Smirnov test, Mann-Whitney U test, and Chi-squared test (3, 4, 9, 21, 30, 35, 37). Nevertheless, we did not find any study which has used the path modeling approach to determine the association of old age and pre-existing comorbidities with the COVID-19 mortality. This was the unique contribution of our approach in modeling COVID-19 mortality.

## Data and Preliminary Methods

We began by providing an overall picture of the dataset that was used for our study. Based on the availability of all the relevant variables, we arrived at a shorter dataset of 117 countries across six regions of the world according to Our World in Data (OWD) (Table 1). The OWD organization compiles global data on pressing global issues. Its data on COVID-19 is widely used by the WHO, the United Nations, and other international organizations. We started with 224 countries in the master list, but we had to cut it down to 117 countries scattered around the world from six broad regions with enough variations as of May 22, 2021. Reasons for cutting down the list of countries were the lack of “COVID-19 test” data, the number of “COVID-19 cases” data, or the death rate by non-communicable disease data. During the revision, we had accessed the latest dataset on September 24, 2021, which was last updated on September 23, 2021. We retrieved the NCD death rate data from World Development Indicator that was updated in 2019^2^.

**Table 1:** List of countries (117).

**sl**	**Asia**	**Europe**	**North america**	**South america**	**Oceania**	**Africa**
1	Azerbaijan	Albania	Belize	Argentina	Australia	Benin
2	Bahrain	Austria	Canada	Bolivia	Fiji	Cote d'Ivoire
3	Bangladesh	Belarus	Costa Rica	Brazil	New Zealand	Ethiopia
4	Bhutan	Belgium	Dominican Republic	Chile	Papua New Guinea	Gabon
5	Cambodia	Bosnia and Herzegovina	El Salvador	Colombia		Gambia
6	China	Bulgaria	Guatemala	Ecuador		Ghana
7	Georgia	Croatia	Jamaica	Paraguay		Kenya
8	Hong Kong	Cyprus	Mexico	Peru		Madagascar
9	India	Denmark	Panama	Uruguay		Malawi
10	Indonesia	Estonia	Trinidad and Tobago			Mauritania
11	Iran	Finland	United States			Morocco
12	Iraq	Germany				Mozambique
13	Israel	Greece				Namibia
14	Japan	Hungary				Nigeria
15	Jordan	Iceland				Rwanda
16	Kazakhstan	Ireland				Senegal
17	Kuwait	Italy				South Africa
18	Laos	Latvia				South Sudan
19	Lebanon	Lithuania				Togo
20	Malaysia	Luxembourg				Tunisia
21	Mongolia	Malta				Uganda
22	Myanmar	Moldova				Zambia
23	Nepal	Netherlands				Zimbabwe
24	Pakistan	Norway				
25	Philippines	Poland				
26	Qatar	Portugal				
27	Saudi Arabia	Romania				
28	Singapore	Russia				
29	South Korea	Serbia				
30	Sri Lanka	Slovakia				
31	Thailand	Slovenia				
32	Timor	Spain				
33	Turkey	Switzerland				
34	United Arab Emirates	Ukraine				
35	Vietnam	United Kingdom				

*Source: Our World in Data*.

The latest dataset we used in our study contains 35 countries from Europe, followed by 35 from Asia, 23 from Africa, 11 from North America, 9 from South America, and 4 from Oceania. It included countries at various stages of development, including least developed countries (LDCs), middle-income countries (MICs), and developed countries. We also used a range of different demographic variables to capture various measures of old age, including median age and the proportion of the population above 65 and 70 years of age. As a proxy for governance, we used the government stringency index, a composite measure of policies enacted to respond to the COVID-19 crisis, and include school and workplace closures and travel bans. Table 2 summarizes the variables that were used in our study.

**Table 2:** Sources of data and variables.

**Name**	**Variable label**	**Conversion**	**Source**
Country code	ISO 3166-1 alpha-3–three-letter country codes		
Country name	Country name		
MAGE	Median age of the population, UN projection for 2020		UN Population Division, World Population Prospects, 2017 Revision
A65	Share of the population that is 65 years and older, most recent year available		World Bank—World Development Indicators, based on age/sex distributions of United Nations Population Division's World Population Prospects: 2017 Revision
A70	Share of the population that is 70 years and older in 2015		United Nations, Department of Economic and Social Affairs, Population Division (2017), World Population Prospects: The 2017 Revision
GDPPC	Gross domestic product at purchasing power parity (constant 2011 international dollars), most recent year available		World Bank – World Development Indicators, source from World Bank, International Comparison Program database
TCPM	Total confirmed cases of COVID-19 per 1,000,000 people	Last available value	COVID-19 Data Repository by the Center for Systems Science and Engineering (CSSE) at Johns Hopkins University
TDPM	Total deaths attributed to COVID-19 per 1,000,000 people	Last available value	COVID-19 Data Repository by the Center for Systems Science and Engineering (CSSE) at Johns Hopkins University
TTPT	Total tests for COVID-19 per 1,000 people	Last available value	National government reports
SI	Government Response Stringency Index: composite measure based on 9 response indicators including school closures, workplace closures, and travel bans, rescaled to a value from 0 to 100 (100 = strictest response)	Average of available values	Oxford COVID-19 Government Response Tracker, Blavatnik School of Government
NCDD	Cause of death, by non-communicable diseases (% of total)		World Development Indicator (WDI)

*Secondary source: Our World in Data and the World Development Indicator*.

*Our cutoff point for data is September 23, 2021, in the cross-section dimension*.

The descriptive statistics for these variables are shown in Table 3. It was observed that the nature of the variables is diverse, which called for a transformation when we adopt them in a modeling framework. Some sort of standardization or log transformation would be helpful in this regard (Table 3). Please find the dataset in Excel and Stata format (.dta) in Supplementary Material. We hope the researchers and readers can easily replicate all the results in Stata by using Path Modeling.

**Table 3:** Descriptive statistics.

**Variable**	**Obs**	**Mean**	**Std.dev**.	**Min**	**Max**
MAGE	117	32.51	9	16.4	48.2
A65	117	10.06	6.52	1.14	27.05
A70	116	6.42	4.52	0.53	18.49
GDPPC	117	22341.7	21206.21	1095.04	116935.6
TCPM	117	51867.66	42811.95	66.48	157075.32
TDPM	117	942.46	961.86	2.17	5970.01
TTPT	117	1122.53	2047.91	8.63	14534.65
SI	117	59.51	9.74	34.39	77.25
NCDD	116	73.02	19.75	25.28	95.17

To eliminate the effect of outlying observations, we used Spearman's rank correlation (Table 4)^3^.

**Table 4:** Spearman rank correlation coefficients.

**Variables**	**(MAGE)**	**(A65)**	**(A70)**	**(GDPPC)**	**(TCPM)**	**(TDPM)**	**(TTPT)**	**(SI)**	**(NCDD)**
MAGE	1.000								
A65	0.915^***^	1.000							
A70	0.901^***^	0.994^***^	1.000						
GDPPC	0.596^***^	0.440^***^	0.433^***^	1.000					
TCPM	0.541^***^	0.470^***^	0.473^***^	0.398^***^	1.000				
TDPM	0.417^***^	0.407^***^	0.412^***^	0.060	0.617^***^	1.000			
TTPT	0.366^***^	0.299^***^	0.298^***^	0.465^***^	0.403^***^	0.059	1.000		
SI	−0.114	−0.241^***^	−0.231^**^	−0.092	0.113	0.153^*^	−0.053	1.000	
NCDD	0.890^***^	0.761^***^	0.740^***^	0.501^***^	0.634^***^	0.465^***^	0.339^***^	0.052	1.000

*** *p <0.01*,

** *p < 0.05*,

* *p < 0.1*.

**Figure 2:**
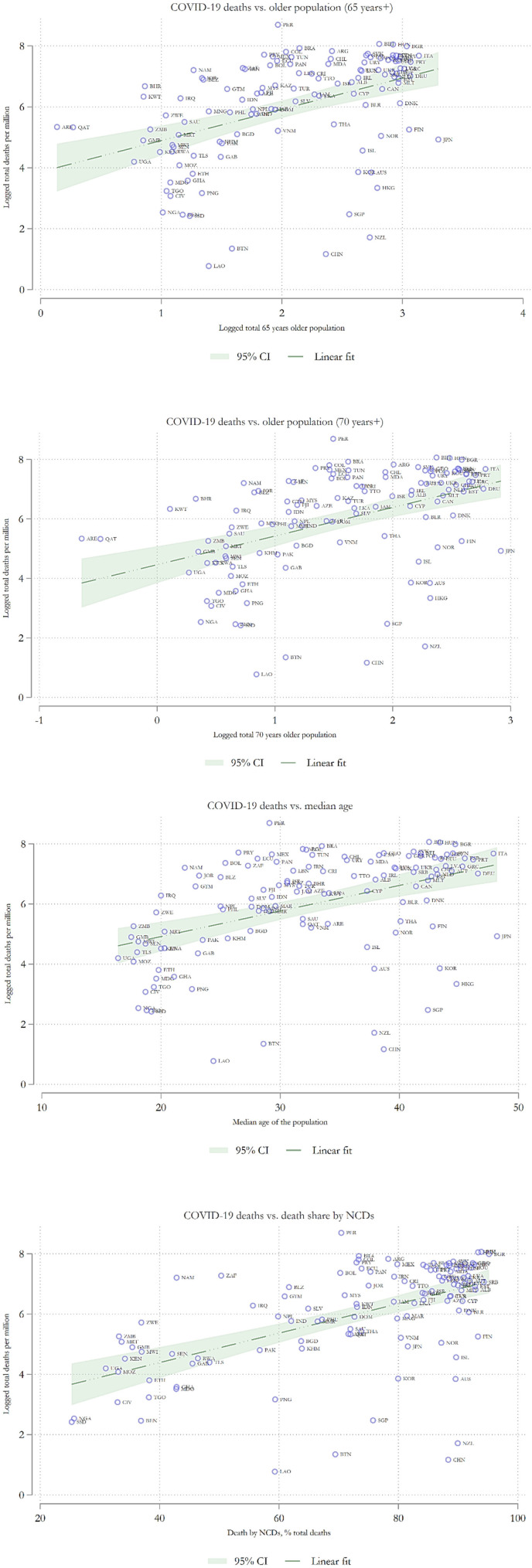
Scatter plots with COVID-19 death as the dependent variable.

The correlation analysis found that median age, age above 65, age above 70, per capita real GDP, transmission, test, and comorbidity *via* non-communicable diseases were highly correlated mainly at a 1% significance level. This information helped formulate suitable models for COVID-19 deaths. We also used two-variable regression-based scatter plots to visualize the pattern of the relationship (Figure 2).

The scatter plots revealed clear positive and linear channels which were prevalent. There was an upward association between logged total deaths per million and logged median age, logged age above 65, and logged age above 70. The upward pattern was also observable when we regressed logged total deaths per million on non-communicable diseases as a percent of total death. Therefore, our scatter plot results had roughly identified two channels of relationship. Nevertheless, these associations were not acceptable without controlling for additional factors. For this reason, a multivariable approach would be more appropriate at this stage to control for other dependencies among the variables.

## Path Model for COVID-19 Death

The path modeling approach has been used in the literature of transmission modeling (1), studies on psychological well-being (14), and other areas, but it has not been used in the context of COVID-19 death modeling which was mainly dominated by multiple regression and simple hypothesis testing. We used two versions of path models: first, the old age model, which focused on the demographic structure of a nation having an impact on COVID-19 deaths after controlling for other factors; second, we used another prominent model, which was known as mortality *via* non-communicable diseases such as cancer, heart diseases, respiratory problems, diabetes. The different types of non-communicable diseases were deadly and were considered an essential factor in raising the probability of death for a COVID-19 patient. The advantage of the path model was that we were able to deal with simultaneity and endogeneity simultaneously without resorting to searching for rare instruments in endogenizing the system. Path modeling is fundamental in the modeling of interdependencies in a structural equation setup. This method is prevalent in social science research and psychology. It is widely used in other interdisciplinary research due to its potential advantages over instrumental variables (IV) technique or Generalized Method of Moments (GMM) estimation.

Model 1. Theoretical path models (base model).


(1a) $$\begin{array}{rcl} TDPM_{i} & = & a_{0}+{a_{1}TCPM}_{i}+\;\varepsilon_{i} \end{array}$$

(1b) $$\begin{array}{rcl} TCPM_{i} & = & \Upsilon_{0}+\Upsilon_{1}TTPT_{i}+\;\Upsilon_{2}SI_{i}+\;\varepsilon_{i} \end{array}$$

(1c) $$\begin{array}{rcl} TTPT_{i} & = & \delta_{0}+\delta_{1}GDPPC_{i}+\;\varepsilon_{i} \end{array}$$

(1d) $$\begin{array}{rcl} SI_{i} & = & \eta_{0}+\eta_{1}GDPPC_{i}+\;\varepsilon_{i} \end{array}$$

**Table:** 

**Parameters**	**Expected signs**
*a*_1_	(+)
*a*_2_	(+)
Υ_1_	(+)
Υ_2_	(+)
δ_1_	(+)
η_1_	(-)

*Source: Based on existing studies*

**Figure:**
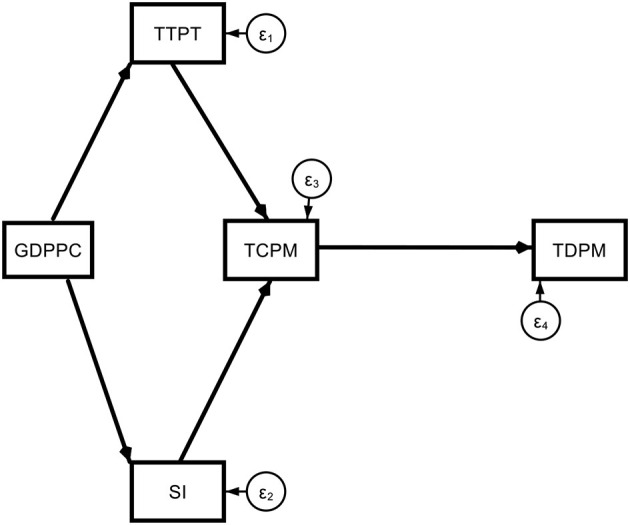


Model 1a. Theoretical path models (base model with regional dummy).


(2a) $$\begin{array}{l} TDPM_{i}=a_{0}+a_{1}TCPM_{i}+\;a_{2}North\_America_{2}+a_{3}Africa_{3}\\ \;+a_{4}Europe_{4}+\;a_{5}Asia_{5}+a_{6}South\_America_{6}\\ \;+a_{7}Oceania_{7}+\varepsilon_{i} \end{array}$$

(2b) $$\begin{array}{rcl} TCPM_{i} & = & \Upsilon_{0}+\Upsilon_{1}TTPT_{i}+\;\Upsilon_{2}SI_{i}+\;\varepsilon_{i} \end{array}$$

(2c) $$\begin{array}{rcl} TTPT_{i} & = & \delta_{0}+\delta_{1}GDPPC_{i}+\;\varepsilon_{i} \end{array}$$

(2d) $$\begin{array}{rcl} SI_{i} & = & \eta_{0}+\eta_{1}GDPPC_{i}+\;\varepsilon_{i} \end{array}$$

**Figure:**
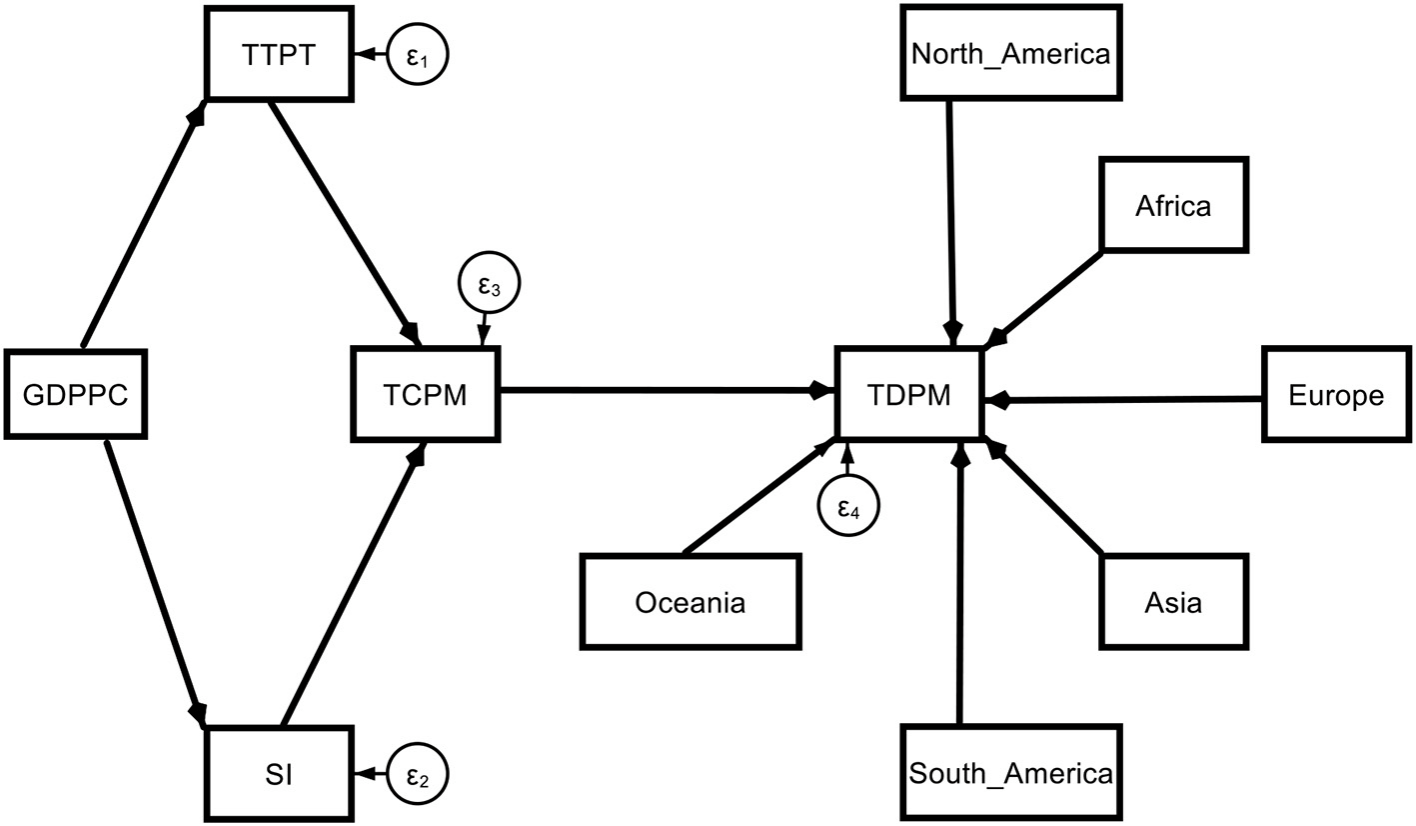


Model 2. Theoretical path models (determinants of COVID-19 deaths *via* old age).


(3a) $$\begin{array}{rcl} TDPM_{i} & = & a_{0}+a_{1}{A65}_{i}+{a_{2}TCPM}_{i}+\;\varepsilon_{i} \end{array}$$

(3b) $$\begin{array}{rcl} TCPM_{i} & = & \Upsilon_{0}+\Upsilon_{1}TTPT_{i}+\;\Upsilon_{2}SI_{i}+\;\varepsilon_{i} \end{array}$$

(3c) $$\begin{array}{rcl} TTPT_{i} & = & \delta_{0}+\delta_{1}GDPPC_{i}+\;\varepsilon_{i} \end{array}$$

(3d) $$\begin{array}{rcl} SI_{i} & = & \eta_{0}+\eta_{1}GDPPC_{i}+\;\varepsilon_{i} \end{array}$$

$$\begin{array}{r} a_{1}>0 \end{array}$$

**Figure:**
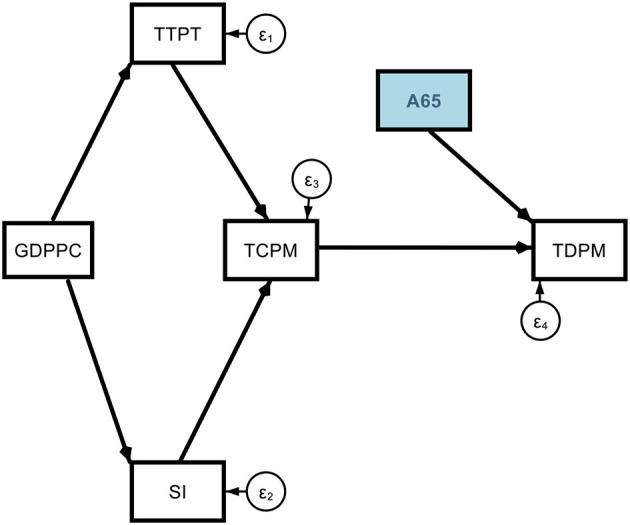


This A65 variable may be replaced by other suitable alternatives such as A70 or MAGE which properly represent the old age hypothesis.

Model 3. Determinants of COVID-19 Deaths *via* non-communicable diseases (NCDD).


(4a) $$\begin{array}{rcl} TDPM_{i} & = & a_{0}+a_{1}NCDD_{i}+{a_{2}TCPM}_{i}+\;\varepsilon_{i} \end{array}$$

(4b) $$\begin{array}{rcl} TCPM_{i} & = & \Upsilon_{0}+\Upsilon_{1}TTPT_{i}+\;\Upsilon_{2}SI_{i}+\;\varepsilon_{i} \end{array}$$

(4c) $$\begin{array}{rcl} TTPT_{i} & = & \delta_{0}+\delta_{1}GDPPC_{i}+\;\varepsilon_{i} \end{array}$$

(4d) $$\begin{array}{rcl} SI_{i} & = & \eta_{0}+\eta_{1}GDPPC_{i}+\;\varepsilon_{i} \end{array}$$

$$\begin{array}{r} a_{1}>0 \end{array}$$

**Figure:**
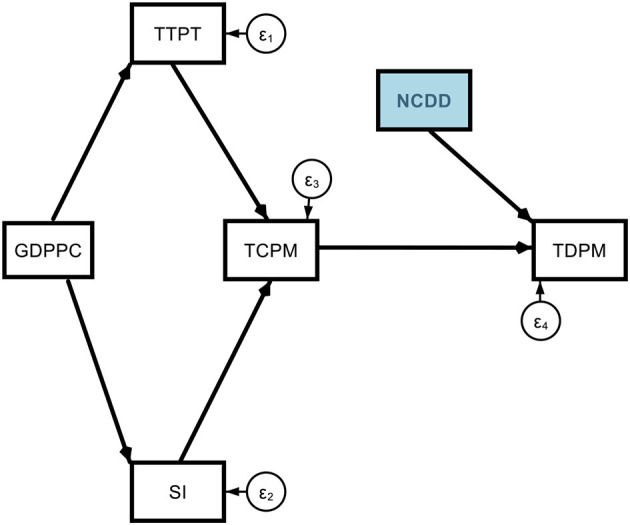


Subscript i = 1, 2, …, 117 represent the country dimension. The arrows represent the direction of the effects, and the parameters represent the magnitude of the effects. The error terms (ε_*i*_) represent the random or unexplained factors in the model. Our main variables of interest are old age and non-communicable disease-related mortality. Other variables were used as additional controls. They represent major direct and indirect channels that affect COVID-19 mortality. The prime determinant is the transmission (TCPM) which itself depends on testing (TTPT), stringency (SI), and per capita GDP (GDPPC).

## Estimated Results and Interpretations

Now we were in a position to estimate the above models in three stages. First, we estimated the base model without dummies for all the countries, and then we estimated the base model with six regional dummies. In this stage, we did not add old age or non-communicable disease mortality. We estimated the old age model without dummies in the second stage, followed by the old age model with regional dummies. The model without regional dummies was meant for the whole world irrespective of region. When we added six regional dummies, they controlled for individual region-specific effects. Third, we estimated the death *via* non-communicable diseases, both without dummies and with dummies. We used the maximum likelihood estimation technique and standardized coefficients for all the models to take care of diverse values except dummy variables. Even though we used six dummies to represent six regions, Oceania was dropped from the final estimation as a reference category to take care of the dummy variable trap. Hence, the estimated dummy coefficients represented relative increments or decrements in the COVID-19 death rate compared with Oceania. If the estimated dummy was positive, it would represent the magnitude by which one region was higher than Oceania. If it was negative, it represented the magnitude by which that region was lower than the benchmark dummy, Oceania. To better understand the whole gamut of effects, we also presented the direct, indirect, and total effects of different shocks to COVID-19 death with their associated significance in standardized forms (Tables 5–10).

**Table 5:** Direct, indirect, and total effects in the base model without dummies.

	**(1)**	**(2)**	**(3)**
	**Direct**	**Indirect**	**Total**
**TTPT**			
GDPPC	0.465^***^	0	0.465^***^
	(4.44)	(.)	(4.44)
**SI**			
GDPPC	−0.0936	0	−0.0936
	(−1.08)	(.)	(−1.08)
**TCPM**			
TTPT	0.408^***^	0	0.408^***^
	(8.31)	(.)	(8.31)
SI	0.137^**^	0	0.137^**^
	(1.73)	(.)	(1.73)
GDPPC	0	0.177^***^	0.177^***^
	(.)	(4.06)	(4.06)
**TDPM**			
TTPT	0	0.252^***^	0.252^***^
	(.)	(6.37)	(6.37)
SI	0	0.0846^**^	0.0846^**^
	(.)	(1.67)	(1.67)
TCPM	0.619^***^	0	0.619^***^
	(8.28)	(.)	(8.28)
GDPPC	0	0.110^***^	0.110^***^
	(.)	(3.56)	(3.56)
*N*	117		

*Source: Own calculation*.

*Standardized beta coefficients; z statistics in parentheses*.

** and *** *represent 5 and 1% significance level, respectively*.

**Table 6:** Direct, indirect, and total effects for the base model with regional dummies.

	**(1)**	**(2)**	**(3)**
	**Direct**	**Indirect**	**Total**
**TTPT**			
GDPPC	0.457^***^	0	0.457^***^
	(4.35)	(.)	(4.35)
**SI**			
GDPPC	−0.0977	0	−0.0977
	(−1.11)	(.)	(−1.11)
**TCPM**			
TTPT	0.427^***^	0	0.427^***^
	(9.48)	(.)	(9.48)
SI	0.141^**^	0	0.141^**^
	(1.78)	(.)	(1.78)
GDPPC	0	0.181^***^	0.181^***^
	(.)	(3.99)	(3.99)
**TDPM**			
TTPT	0	0.185^***^	0.185^***^
	(.)	(3.97)	(3.97)
SI	0	0.0613^*^	0.0613^*^
	(.)	(1.56)	(1.56)
TCPM	0.434^***^	0	0.434^***^
	(4.17)	(.)	(4.17)
GDPPC	0	0.0787^***^	0.0787^***^
	(.)	(2.84)	(2.84)
Asia	0.0126	0	0.0126
	(0.39)	(.)	(0.39)
Africa	0.0693^**^	0	0.0693^**^
	(1.67)	(.)	(1.67)
North America	0.188^***^	0	0.188^***^
	(3.33)	(.)	(3.33)
South America	0.556^***^	0	0.556^***^
	(6.69)	(.)	(6.69)
Europe	0.367^***^	0	0.367^***^
	(3.99)	(.)	(3.99)
*N*	116		

*Source: Own calculation*.

*Standardized beta coefficients; z statistics in parentheses*.

*, **, and *** * represent 10, 5, and 1% significance level, respectively*.

**Table 7:** Direct, indirect, and total effects determinants of COVID-19 deaths *via* A65.

	**(1)**	**(2)**	**(3)**
	**Direct**	**Indirect**	**Total**
**TTPT**			
GDPPC	0.465^***^	0	0.465^***^
	(4.43)	(.)	(4.43)
**SI**			
GDPPC	−0.0920	0	−0.0920
	(−1.06)	(.)	(−1.06)
**TCPM**			
TTPT	0.410^***^	0	0.410^***^
	(8.35)	(.)	(8.35)
SI	0.135^**^	0	0.135^**^
	(1.70)	(.)	(1.70)
GDPPC	0	0.178^***^	0.178^***^
	(.)	(4.06)	(4.06)
**TDPM**			
TTPT	0	0.232^***^	0.232^***^
	(.)	(6.30)	(6.30)
SI	0	0.0761^*^	0.0761^*^
	(.)	(1.60)	(1.60)
TCPM	0.566^***^	0	0.566^***^
	(7.25)	(.)	(7.25)
GDPPC	0	0.101^***^	0.101^***^
	(.)	(3.55)	(3.55)
A65	0.155^*^	0	0.155^*^
	(1.58)	(.)	(1.58)
*N*	117		

*Source: Own calculation*.

*Standardized beta coefficients; z statistics in parentheses*.

*, **, and *** *represent 10, 5, and 1% significance level, respectively*.

**Table 8:** Direct, indirect, and total effects of the determinants of COVID-19 deaths *via* A65 with regional dummies.

	**(1)**	**(2)**	**(3)**
	**Direct**	**Indirect**	**Total**
**TTPT**			
GDPPC	0.457^***^	0	0.457^***^
	(4.35)	(.)	(4.35)
**SI**			
GDPPC	−0.0977	0	−0.0977
	(−1.11)	(.)	(−1.11)
**TCPM**			
TTPT	0.427^***^	0	0.427^***^
	(9.48)	(.)	(9.48)
SI	0.141^**^	0	0.141^**^
	(1.78)	(.)	(1.78)
GDPPC	0	0.181^***^	0.181^***^
	(.)	(3.99)	(3.99)
**TDPM**			
TTPT	0	0.183^***^	0.183^***^
	(.)	(4.06)	(4.06)
SI	0	0.0606^*^	0.0606^*^
	(.)	(1.56)	(1.56)
TCPM	0.429^***^	0	0.429^***^
	(4.27)	(.)	(4.27)
GDPPC	0	0.0778^***^	0.0778^***^
	(.)	(2.88)	(2.88)
Asia	0.0386	0	0.0386
	(0.82)	(.)	(0.82)
Africa	0.113^*^	0	0.113^*^
	(1.64)	(.)	(1.64)
North America	0.195^***^	0	0.195^***^
	(3.26)	(.)	(3.26)
South America	0.564^***^	0	0.564^***^
	(6.82)	(.)	(6.82)
Europe	0.314^***^	0	0.314^***^
	(2.93)	(.)	(2.93)
A65	0.107	0	0.107
	(1.05)	(.)	(1.05)
*N*	116		

*Source: Own calculation*.

*Standardized beta coefficients; z statistics in parentheses*.

*, **, and *** *represent 10, 5, and 1% significance level, respectively*.

**Table 9:** Direct, indirect, and total effects of the determinants of COVID-19 deaths *via* NCDD without dummies.

	**(1)**	**(2)**	**(3)**
	**Direct**	**Indirect**	**Total**
**TTPT**			
GDPPC	0.457^***^	0	0.457^***^
	(4.35)	(.)	(4.35)
**SI**			
GDPPC	−0.0977	0	−0.0977
	(−1.11)	(.)	(−1.11)
**TCPM**			
TTPT	0.427^***^	0	0.427^***^
	(9.48)	(.)	(9.48)
SI	0.141^**^	0	0.141^**^
	(1.78)	(.)	(1.78)
GDPPC	0	0.181^***^	0.181^***^
	(.)	(3.99)	(3.99)
**TDPM**			
TTPT	0	0.236^***^	0.236^***^
	(.)	(6.02)	(6.02)
SI	0	0.0782^**^	0.0782^**^
	(.)	(1.65)	(1.65)
TCPM	0.554^***^	0	0.554^***^
	(6.44)	(.)	(6.44)
GDPPC	0	0.100^***^	0.100^***^
	(.)	(3.40)	(3.40)
NCDD	0.132^*^	0	0.132^*^
	(1.62)	(.)	(1.62)
*N*	116		

*Source: Own calculation*.

*Standardized beta coefficients; z statistics in parentheses*.

*, **, and *** *represent 10, 5, and 1% significance level, respectively*.

**Table 10:** Direct, indirect, and total effects of the determinants of COVID-19 deaths *via* NCDD with regional dummies.

	**(1)**	**(2)**	**(3)**
	**Direct**	**Indirect**	**Total**
**TTPT**			
GDPPC	0.457^***^	0	0.457^***^
	(4.35)	(.)	(4.35)
**SI**			
GDPPC	−0.0977	0	−0.0977
	(−1.11)	(.)	(−1.11)
**TCPM**			
TTPT	0.427^***^	0	0.427^***^
	(9.48)	(.)	(9.48)
SI	0.141^**^	0	0.141^**^
	(1.78)	(.)	(1.78)
GDPPC	0	0.181^***^	0.181^***^
	(.)	(3.99)	(3.99)
**TDPM**			
TTPT	0	0.164^***^	0.164^***^
	(.)	(3.69)	(3.69)
SI	0	0.0544^*^	0.0544^*^
	(.)	(1.52)	(1.52)
TCPM	0.386^***^	0	0.386^***^
	(3.79)	(.)	(3.79)
GDPPC	0	0.0699^***^	0.0699^***^
	(.)	(2.71)	(2.71)
Asia	0.0630	0	0.0630
	(1.16)	(.)	(1.16)
Africa	0.238^**^	0	0.238^**^
	(1.79)	(.)	(1.79)
North America	0.218^***^	0	0.218^***^
	(3.35)	(.)	(3.35)
South America	0.594^***^	0	0.594^***^
	(7.56)	(.)	(7.56)
Europe	0.358^***^	0	0.358^***^
	(3.75)	(.)	(3.75)
NCDD	0.213^*^	0	0.213^*^
	(1.59)	(.)	(1.59)
*N*	116		

*Source: Own calculation*.

*Standardized beta coefficients; z statistics in parentheses*.

*, **, and *** *represent 10, 5, and 1% significance level, respectively*.

Model 1. estimated path model (base model without a dummy).

**Figure:**
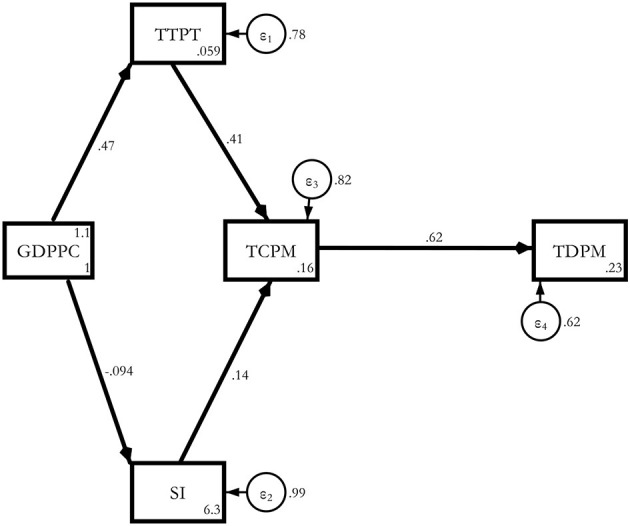


Without taking any demographic or comorbidity variables, a one *SD* increase in transmission led to a 0.62 standard deviation increase in COVID-19 deaths^4^.

The total effects were statistically significant *via* all the channels of testing (0.252), stringency (0.0846), transmission (0.619), and per capita GDP (0.11), with the highest effect coming from the transmission makes intuitive sense. This result was valid for the whole sample (Table 5).

Model 1a. Estimated Base model with Regional Dummies.

**Figure:**
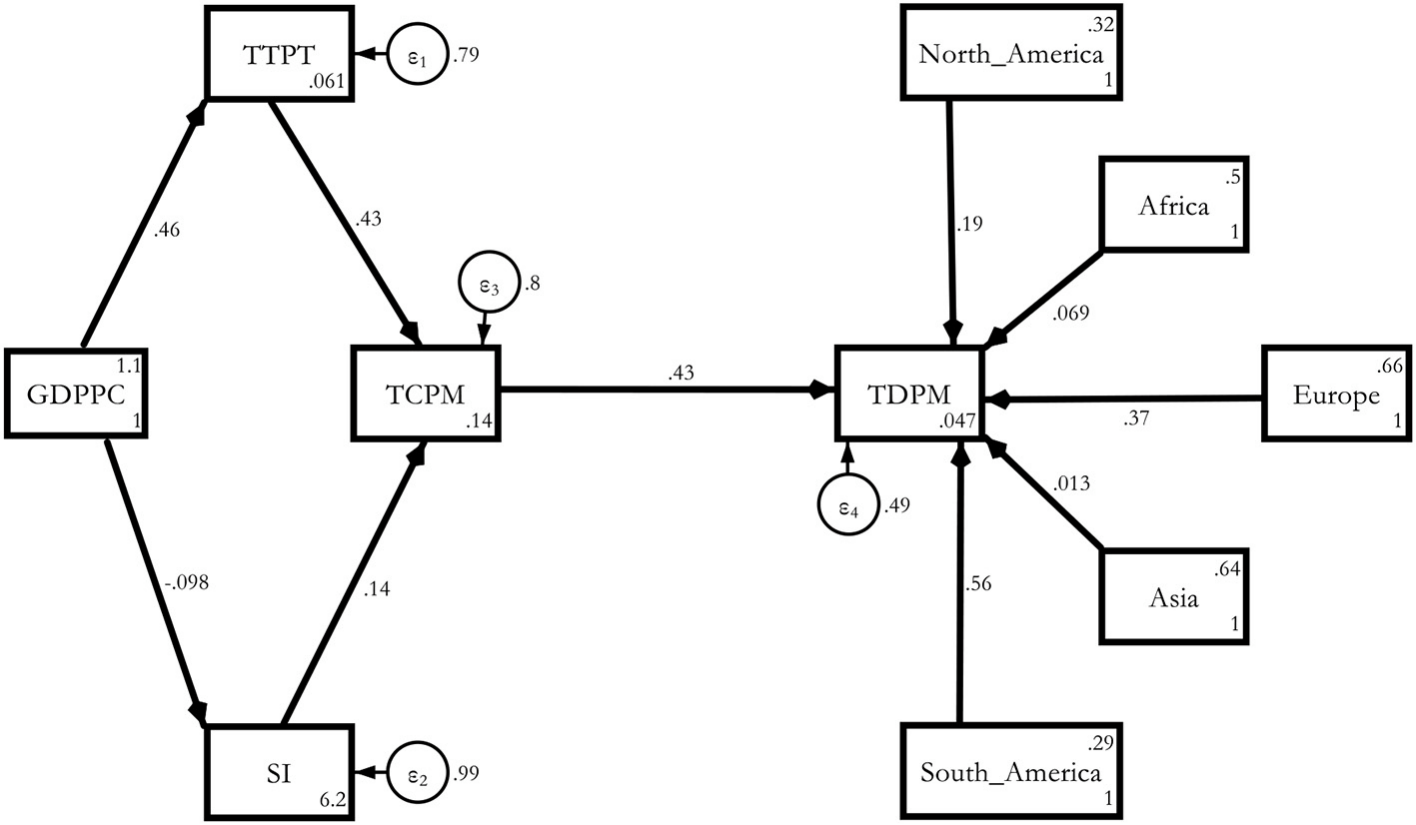


The regional effect was the most severe for South America (0.556), followed by Europe (0.367), North America (0.188), Africa (0.0693), and Asia (0.0126) compared with Oceania. These results held without any old age or non-communicable disease channel in the model (Table 6).

Model 2. Estimated Determinants of COVID-19 deaths *via* A65.

**Figure:**
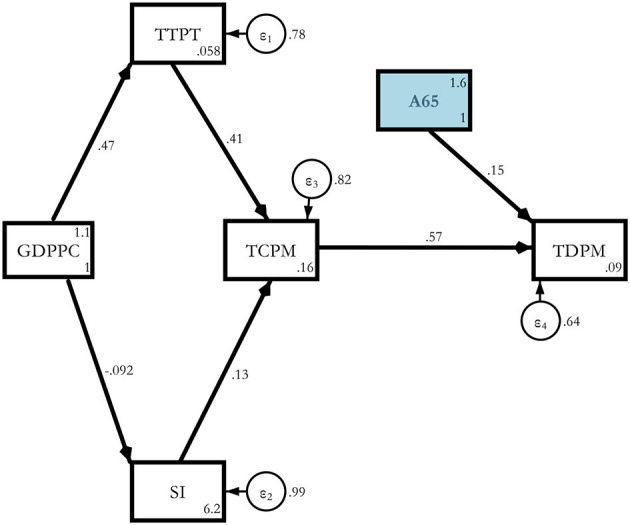


Here, we introduced old age in the model as an additional channel. When we introduced A65 as an additional regressor, its magnitude turned out to be 0.155 with a 10% significance level. This was for the whole sample of 117 countries of the world (Table 7).

Model 2a. Estimated determinants of COVID-19 deaths *via* A65 with regional dummies.

**Figure:**
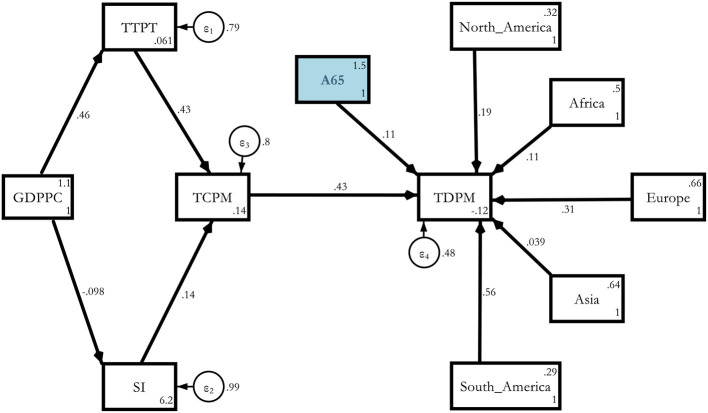


When we ran the path again with A65 and regional dummies, South America turned out to be the worst affected followed by Europe, North America, Africa, and the severity was the lowest in Asia compared with Oceania (Table 8). However, the A65 coefficient lost its significance. As a cross-check, we also ran the same model with MAGE and found that the coefficient (0.12) was significant at 10% for the 117 countries for the overall model without dummies and 0.173 with a 5% significance level for the regional model with dummies^5^.

Now we were able to present the result from our third model to show the impact of non-communicable disease mortality on COVID-19 death. We started with the global model without regional dummies (Table 9).

Model 3. Estimated determinants of COVID-19 deaths *via* NCDD without dummies.

**Figure:**
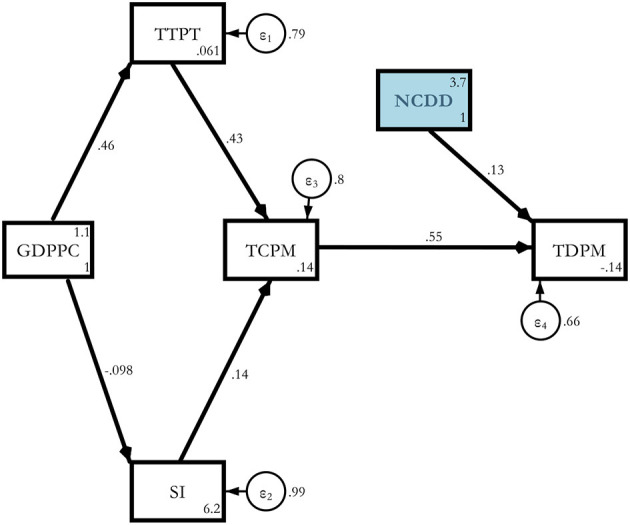


The result indicated that a 1% increase in non-communicable disease mortality raised COVID-19 death by 0.132 with an *SD* at a 5% significance level for the whole sample of 117 countries (Table 9). Now, we switched to the regional model for non-communicable diseases to check whether the global result was robust to regional variation (Table 10).

Model 3a. Estimated Determinants of COVID-19 deaths *via* NCDD with regional dummies.

**Figure:**
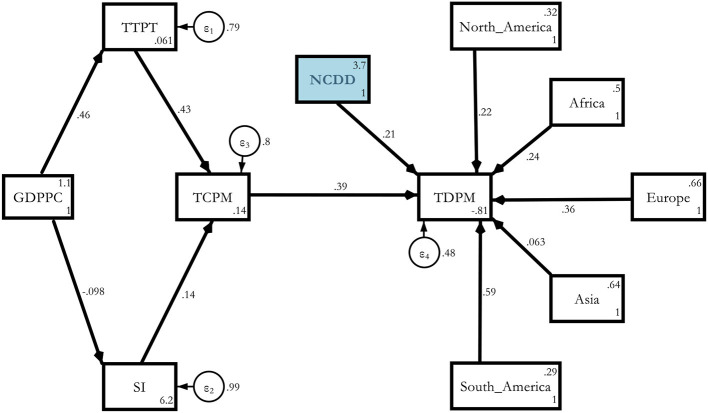


The severity of the effect was even worse because the magnitude of the effect now turned out to be 0.213 compared with the purely global model, which was just 0132 at the same 5% level of significance. The regional severity was the worst in South America (0.594), followed by Europe (0.358), Africa (0.238), North America (0.218), and Asia (0.063) compared with Oceania (Table 10)^6^. This meant that the NCD channel became more prominent after controlling for individual region-specific heterogeneity.

In Table 11 we provided a combined summary of the determinants of COVID-19 deaths worldwide for September 23, 2021, using the base model and two other models of our choice.

**Table 11:** Determinants of COVID-19 death at a glance (dependent variable: total death per million of population).

**Indep. Var (only selected var)**	**1 (base model without a dummy)**	**1a (base model with dummy)**	**2 (old age model without a dummy)**	**2a (old age model with dummy)**	**3 (Non-comm without a dummy)**	**3a (Non-comm with dummy)**
TCPM	(+)^***^	(+)^***^	(+)^***^	(+)^***^	(+)^***^	(+)^***^
Old age			(+)^*^	(+)		
Non-comm					(+)^*^	(+)^*^
Asia		(+)		(+)		(+)
Africa		(+)^**^		(+)^**^		(+)^**^
N. America		(+)^***^		(+)^***^		(+)^***^
S. America		(+)^***^		(+)^***^		(+)^***^
Europe		(+)^***^		(+)^***^		(+)^***^

*, **, and *** *represent 10, 5, and 1% significance level, respectively. Oceania is dropped as a reference region. + and – sign represent the direction of effect*.

Summarizing the results the results, we found that the base model without dummy turned out to be sensible and identified transmission (TCPM) as the significant determinant. The result was held when we moved toward a dummy model with South America, Europe, and North America identified as the worst affected areas. When we used A65 without the regional dummies, the coefficient was significant at the 10% level and lost its significance when we controlled for regional variation. However, the result seemed to be strongly supported at the overall and regional level if we used MAGE in place of A65 or A70. When we ran the same old age model with dummies, we found that wide variation across regions was profound with no significant impact on Asia. In contrast, North America, South America, and Europe were the worst affected areas with no significant age effect in Asia compared with Oceania. Then we turned our attention to the non-communicable disease channel and found it a significant determinant of COVID-19 death without regional dummies at a 10% level. Finally, we ran the same model with regional dummies for non-communicable diseases and found an insignificant result for Asia but highly significant for South America, Europe, North America, and Africa (Table 11). This meant that regional severity was a big issue both for old age and non-communicable disease during the COVID-19 outbreak.

## Further Discussion

The sheer scale of the pandemic that has taken millions of lives throughout the globe presents a lingering mortality puzzle that is yet to be solved. It is a question whether what can explain the divergence in mortality figures across different regions with varied demographic structures, climates, and institutional contexts. Thus far, many variables have been studied, including the possible role of social and ethnic fragmentation, corruption, the relative immunity of populations, and the extent to which experience with previous epidemics may influence mortality deaths.

In the early days of the pandemic, it was argued that the COVID-19 virus had a temperate climate bias, but this theory was shed when the virus spread the specter of death to warmer climates. It was also contended that proximity to China would impact spread and ultimately mortality, but this was not found to be the case with countries like Thailand, South Korea, and Taiwan reporting relatively low mortality rates, as pointed out by Mandelman (39). The role of female leadership and the nature of political institutions have also been considered possible determinants of mortality incidence. New Zealand is a striking case in point that has been used to illustrate the role of female leadership in containing the virus. The government undertook flight bans and nationwide lockdowns early on to avert widespread community transmission. However, studies such as that by Windsor et al. (40) and Bosancianu et al. (41) dispel such theories that essentialize leadership based on gender as subject to selection bias and not standing their ground when tested using the latest empirical data.

Since the start of the pandemic, the research battery does not provide any conclusive direction as to any single set of factors, such as demographic, climatic, socio-economic, or political, that drive mortality deaths. For instance, take the case of Peru, which has the highest per-capita mortality rate from the virus; this, despite implementing lockdowns and enforcing near-universal mask usage (39). The same goes for Argentina, which has also recorded high mortality rates despite strict social distancing policies. In contrast, as Mandelman (39) notes, the mortality rate in Nigeria was negligible despite no enforcement of mask usage. Uruguay is another example of a country with negligible mortality rates despite relatively simple measures to contain the virus. Brazil is also reported to have witnessed significantly lower mortality cases than Peru despite patchy social distancing measures and a deprioritizing of the importance of the virus at the federal level.

The study of Bosancianu et al. (41) have tested a wide range of political and social variables, including political institutions, state capacity, independence of media, and regime types. The role of free and independent media in influencing mortality has been well-researched. It dates back to the famine counts that Dreze and Sen (42) discussed using a comparative analysis of India and China. However, there is only inconclusive evidence concerning the role that democratic institutions have played in either exacerbating or mitigating the death toll from the pandemic. Carpenter (43) cogently argues that public health crises are an arena with less divergence across countries with different institutional arrangements due to the immediate ‘moral claims' attached to health than other concerns relating to the environment in other areas. This provides a compelling explanation of why there is little in the way of significant, distinguishable differences in the incidence of mortality from COVID-19 across different regime types.

The findings of Bosancianu et al. (41) also suggest how fluid the associations are between these variables with the strength of the association changing over time, thus reflecting changes in policy responses which are, in turn, contingent on changes in transmission spread of a mutating virus. The authors found that institutional trust is negatively associated with mortality rates, albeit with the caveat that there is limited data that can be used to proxy measures of trust. Government effectiveness as measured by public perception of public services quality was also correlated with mortality rates in this study. However, the role of the overall distribution of power and the extent to which power is centralized or decentralized was found to have an ambiguous association with mortality.

Returning to our study, the findings from the path model were 2-fold: (1) old age and comorbidity stood out as having a clear and direct causal association with COVID-19 mortality; (2) in addition to these demographic and health-related causes, there were clear pathways associated with geographical location with a greater intensity of deaths in South America, Europe, and flowed by North America. Although governance and policy matter, the channels of causation were indirect. As the old age model suggested, Africa has a relatively low regional intensity of COVID-induced mortality than other regions like Europe. This was consistent with the demographic structure of the population of Africa, where the median age was the lowest compared with other regions of the globe. The non-communicable disease model also suggested that Africa is a continent where communicable disease constituted a more significant disease burden than NCDs.

However, there were some paradoxical results. Based on the estimated base model with regional dummies, the regional effect was the most severe for South America, followed by Europe. However, when the path was reran using the proportion of the population that was 65 years or older as well as other variables like median age, South America was the worst affected followed by Europe, Africa, and Asia has the lowest severity. This was intriguing considering that the median age of South America was considerably lower than Europe. Moreover, South America and Asia had similar median ages. These findings suggested a confluence of variables and pathways that impact mortality. However, old age was one such path that we found to be statistically significant; we could negate the complexity and the importance of other factors influencing mortality outcomes.

In our path model, the government response stringency index did not have a direct impact on mortality. However, this did not mean that preventive measures like lockdowns, social distancing, and widespread mask usage were unnecessary. Instead, this could mean that apart from stringency, other forms of public response and, in particular, public services like personal protective equipment and hospital beds could have a more significant and more direct impact on mortality, as Liang et al. (44) had reported in their study. Vietnam is a striking example of a country that has developed a vast public health architecture, including a surveillance system and a public health emergency operations center. As the OWD reported, Vietnam was also a country with the benefit of experience and learning by doing in managing the SARS epidemic.

Another notable example that was widely reported in the media in China was the speedy construction of makeshift hospitals to treat infected patients. These ‘Fangcang' temporary hospitals had been a crucial part of the public health response in China and involved the speedy conversion of stadiums and exhibition centers into health care facilities (45). On the other hand, Italy was ill-equipped to deal with the surge of COVID-19 cases. This outcome might have come about from long-standing spending cuts for healthcare in addition to patchwork, preventive responses that were not quick or comprehensive enough (46). A similar set of problems arose in the United Kingdom. As cases surged there and to cope with hospital demand, infected patients were discharged to care homes that did not have the proper means to contain the spread of infections (47).

These cases, as mentioned above, showed the importance of government responsiveness and not necessarily stringency measures alone. Some countries responded on time and were fluid in the measures and policies they took on. Preventive measures such as lockdowns and social distancing were part of the response. However, ensuring adequate treatment facilities with adequate medical supplies was also equally important. The lack of a powerful and direct impact of the stringency index in our study might be due to the importance of other aspects of government effectiveness that were not measured in this stringency index.

## Conclusion and Policy Suggestions

Coronavirus disease 2019-induced mortality remains the most pressing concern globally. It has challenged all countries in varying degrees. We have identified two channels of COVID-19 death globally, namely, old age and non-communicable disease-related comorbidity are identified as the two prime determinants of COVID-19 deaths. We developed two path models using data for COVID-19 transmission, test, death, per capita real GDP, and stringency as additional control variables. We tested them for 117 countries using the cross-section data from OWD. The models were ran for the whole sample of 117 countries without the regional dummies and with the regional dummies. We found that the path for old age and non-communicable disease-related mortality toward COVID-19 were prominent. Therefore, we could safely contend that for the whole sample of 117 countries, both the old age model and non-communicable disease model significantly impacted the COVID-19 death rate. In contrast, the models had rightly identified South America as the worst-hit area, followed by Europe, North America, Africa, and Asia. This was despite the surge in cases in specific countries within Asia like India, for instance, due to the spread of the Delta variant.

The result was valid when we use Oceania as the benchmark or reference region. The study suggested that senior citizens and people suffering from comorbidity *via* non-communicable diseases like cancer, diabetes, heart diseases, and respiratory diseases should receive priority and subsidization in vaccination, development assistance, and other government support. Particular attention should be given to the affected people from South America, Europe, followed by North America at this stage. However, the results should be analyzed with caution, given that it only captured the data observed on September 23, 2021. Although the pattern of variants is an essential aspect of COVID-19, it remained as one of the study limitations. It could not identify the differences among the variants and could not conclude how the Delta variant may play a role in the context. Our study used a cross-sectional dataset which did not allow us to distinguish among different variants. Instead, it captured the influences of all types of variants that have gripped the world during the period under consideration.

## Data Availability Statement

The original contributions presented in the study are included in the article/Supplementary Material, further inquiries can be directed to the corresponding author.

## Author Contributions

All authors listed have made a substantial, direct and intellectual contribution to the work, and approved it for publication.

## Conflict of Interest

The authors declare that the research was conducted in the absence of any commercial or financial relationships that could be construed as a potential conflict of interest.

## Publisher's Note

All claims expressed in this article are solely those of the authors and do not necessarily represent those of their affiliated organizations, or those of the publisher, the editors and the reviewers. Any product that may be evaluated in this article, or claim that may be made by its manufacturer, is not guaranteed or endorsed by the publisher.

## Footnotes

^1^Deep red color indicates high concentration of COVID-19 death or mortality in South America, Europe, followed by North America. Asia, Africa, and Oceania seem to be safer with light color.

^2^We are grateful to an anonymous referee for enlightening us with the issue of omission of some countries and we have updated the list with a larger set of countries.

^3^We are grateful to an anonymous referee for pointing out that reporting Pearson is redundant in this case and only reporting Spearman's rank correlation coefficients is sufficient.

^4^The difference between Model 1 and Table 5 is that Table 5 represents total effect, direct effect, and indirect effect whereas Model 1 shows only direct effects with arrows representing the direction of the effects.

^5^The estimated paths with MAGE are not reported due to space constraint but the result is available upon request. The discrete cut-off age by 60 or 70 is becoming redundant. This could be the end result of Delta variant which also affects people from any age.

^6^As an additional robustness checking we had modified the path model with both old age and comorbidity in the same model for global data for 117 countries and also for model with regional dummies and found that our hypothesis was supported. For countries as a whole the result held at 10% level only for A65 and for the regional model the result held for 10% only for NCD death. This meant that if a person is old and also suffers from noncommunicable disease, that person has high probability of death due to COVID-19. The result was not reported due to space constraint.
